# A case report of black swan (*Cygnus atratus*) died from gastric perforation and secondary infection resulting from ingestion of cloth—like foreign material

**DOI:** 10.3389/fvets.2025.1608317

**Published:** 2025-09-02

**Authors:** Xianqin Jiao, Haoqian Wang, Yongchao Yan, Cossi Remi Codjia, Xinwei Wang

**Affiliations:** College of Veterinary Medicine, Henan Agricultural University, Zhengzhou, China

**Keywords:** black swan, intestinal obstruction, bacterial infection, gastric perforation, case report

## Abstract

In this case, the cause of death of black swan was determined by investigating the daily feeding situation of black swan and combining clinical diagnosis, pathological anatomy, PCR detection and other methods. Clinical autopsy results showed that the dead black swan was dehydrated and emaciated with its stomach filled with large amounts of fine sand and black cloth. The main pathological manifestations were peritonitis, air sac inflammation, perihepatic inflammation and necrosis, pericarditis, and dendritic pancreatic hemorrhage. Simultaneously *Shigella boydii*, *Escherichia coli* and *Enterococcus faecalis* were isolated from black swan bodies. The clinical evidence above showed that the swan died of ingesting human fabric, which blocked its stomach and caused a perforation, followed by a secondary bacterial infection. It is the first reported swan death caused because of eating human-cloth waste, suggesting that humans still need to work hard to protect the environment and care for animals.

## 1 Introduction

The black swan (*Cygnus atratus*), a large waterfowl of the genus Cygnus, family Anatidae, order Anseriformes, predominantly inhabits freshwater lakes, swamps, and estuaries (1–3). Its diet mainly comprises aquatic plants and algae, with occasional consumption of aquatic insects or small fish. Black swans, as precious wild waterfowl, possess significant ornamental value (4, 5). Black swans have robust constitutions, enabling them to resist high temperatures and severe cold effectively, thus making them less likely to contract diseases. Currently, there are relatively few reports on black swan cases worldwide. The main types of reported diseases include: parasitic infection (5, 6), viral diseases (7–9), bacterial diseases (3, 10–16), mixed—infection (17, 18), and non—infection diseases (Chronic T-cell Lymphocytic Leukemia) (19), Cervical dorsal spondylosis (20) and goose gout (21). So far, there are no documented cases of swans dying from eating cloth strips for reference. This article reported for the first time a case of one black swan that died of obstruction of cloth strips in gizzard resulting in gastric perforation and secondary infection.

## 2 Case presentation

**Figure 1:**
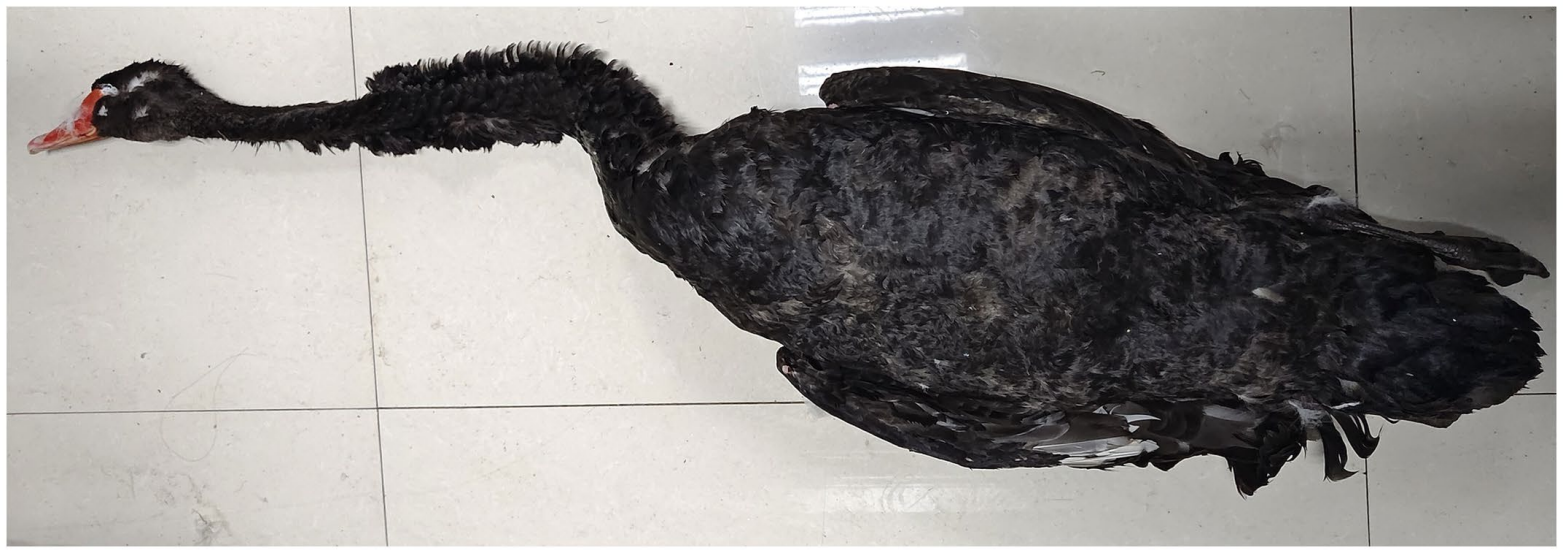
The dead black swan.

On September 23, 2023, a swan breeder from Henan Agricultural University in Henan Province, China, contacted me to report a death case of a black swan inhabiting in the North Lake of the university campus, and requested help in determining what caused the swan to die so that some measures should be taken to protect the remaining black swans in the lake. Due to my being occupied with an out—of—town academic meeting assignment at the time, a concise inquiry was made via telephone regarding the status of the deceased swan and the other swans [it was reported on the phone that this adult black swan was found dead during the morning feeding. It was emaciated and relatively lightweight. Usually, swans were fed with green vegetables and corn, and they would also freely forage for waterweeds, fish, shrimp in the lake, as well as food fed by humans. All swans had no previous disease history. The surrounding environment is favorable for black swans’ survival, and it had been vaccinated against gosling plague, paramyxovirus disease, and highly pathogenic influenza. The other black swans in the lake were all normal (Figure 1)]. Therefore, It was proposed that the carcass of the deceased black swan be preserved by means of freezing to enable subsequent clinical diagnosis and testing. Post-mortem examination and relative clinical survey were not performed until September 26, 2023. The final diagnosis was that the swan died due to the abnormal blockage of its digestive tract caused by swallowing a piece of fabric from human clothing.

## 3 Epidemiological investigation

12 black swans including the deceased one were raised in the artificial university campus lake with good water quality. These swans predominantly roosted on an island within the lake at night (Supplementary Figure 1). No other individuals apart from the swan keeper could contact with the birds. On the other hand, there were no other domestic fowls or wild birds apart from sparrows on the island. The keeper regularly fed corn bran and green vegetables to the black swans. All the black swans had been vaccinated against gosling plague, paramyxovirus disease, and highly pathogenic influenza, with each vaccine administered twice.

### 3.1 Clinical symptoms

Except for the deceased black swan, no obvious clinical changes were observed in the other black swans in the lake. No diseases or deaths in the area where these black swan inhabited were seen among other birds either. Upon investigation, it was found that the black swan did not exhibit any signs of fighting behavior prior to its death. The results of the clinical examination indicated that there were no obvious external traumas on the body surface of the deceased black swan (Figure 1). The oral, nasal, and anal regions were relatively clean. However, its body weight was on the lighter side, showing extreme emaciation. The pectoral muscles presented signs of dehydration and atrophy, and the protrusion of the xiphoid cartilage of the chest could be felt upon palpation. Additionally, its toe—sole area appeared shriveled and lacked luster, demonstrating signs of dehydration.

### 3.2 Clinical necropsy

**Figure 2:**
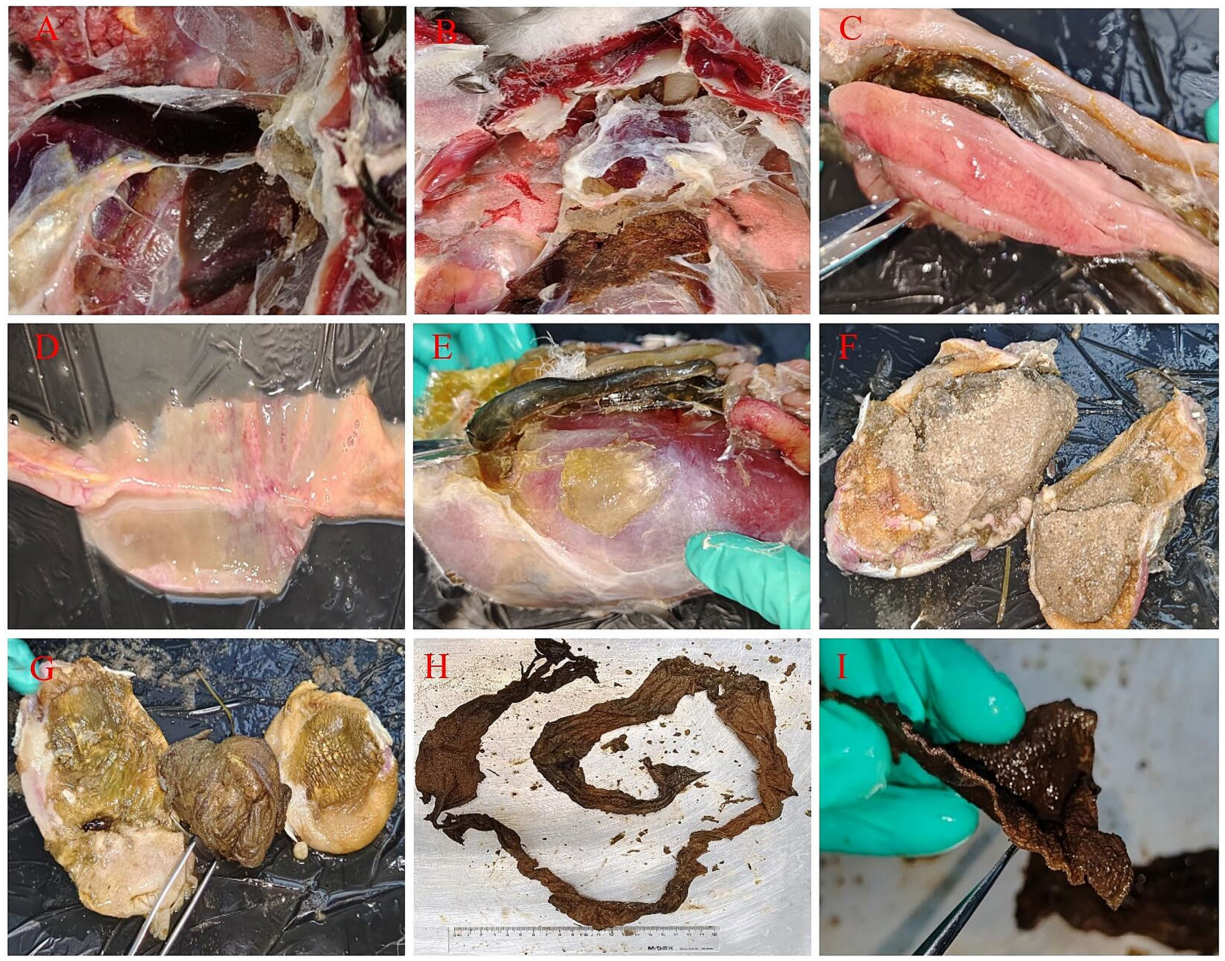
Necropsy results of the black swan. **(A)** The liver showed white necrotic foci with inflammatory exudates around them (congestion necrosis, perihepatic inflammation, inflammatory exudate attached to the capsule); **(B)** pericarditis (with a large amount of fibrinous exudate attached to the pericardium) and gastric contents leaked into the abdominal cavity; **(C)** dendritic hemorrhaging of the pancreas; **(D)** intestinal hemorrhaging; **(E)** enlarged proventriculus (extremely enlarged glandular stomach and muscular stomach); **(F)** fine sand in the gizzard; **(G)** gastric perforation, ulcers and foreign objects in the stomach; **(H,I)** cloth strips in the stomach (the undecomposed human T-shirt-shaped cloth strip was about one meter long, and its section shows cloth fibers).

The necropsy results showed (Figure 2) significant distension in both the gizzard and proventriculus exhibited, with a notable perforation in the proventriculus. Peritonitis together with air sac inflammation were observed. The liver appeared enlarged and congestion, with signs of necrosis and perihepatitis, its surface covered in fibrinous exudate. The pancreas displayed dendritic hemorrhaging, and there were pronounced symptoms of pericarditis, while alterations in the lungs were comparatively less significant. Upon incision of the gizzard and proventriculus, a substantial quantity of fine sand and aggregates of black solid material were identified within the proventriculus. Further examination indicated that the black substance was determined to be elongated, undecomposed human T-shirt-shaped cloth strip with nearly one meter in length. Additionally, an ulcer was noted at the junction of the gizzard and proventriculus, accompanied by evident intestinal hemorrhaging. Pathological anatomical changes suggested that the swan died from bacterial infection caused by abnormal gastrointestinal obstruction.

## 4 Laboratory bacteriological examination

The liver tissue samples of the dead black swan were collected and processed. Then blood agar plates were utilized to conduct the isolation, purification, and cultivation of bacteria in the laboratory. Individual colonies on blood agar nutrient plates were further isolated and cultured through using LB liquid medium. Subsequently, the TIANamp Bacteria DNA Kit (TIANGEN, China; catalog number: DP302) was employed to extract the genomic DNA of the isolated bacteria. PCR amplification was carried out by using the 16S rRNA universal primers (22) (27F: AGAGTTTGATCCTGGCTCAG, 1492R: CTACGGCTACCTTGTTACGA). Then the PCR-amplification products were detected by 1% agarose gel electrophoresis, and dispatched to Shanghai Biotech Co., Ltd. for further being sequenced. In the end, the sequences results were subjected to sequence alignment analysis using the BLAST tool on the NCBI website to determine the genus and species of the bacteria. Additionally, the MEGA7.0.14 software was utilized to construct a phylogenetic tree based on the 16S rRNA sequence via the neighbor—joining method for further analysis (see Table 1).

**Table 1:** 16S rRNA reference strains.

GenBank	Strain name	Country	Time
MT573569	1,044	China	2020
OP431821	PM11	Italy	2022
HQ805622	ELU0156-T284-S-NIPCRAMgANa_000140	USA	2012
OP491971	CD-H-10	South Korea	2022
MN252109	KR 23	India	2019
NR026331	ATCC 29903	USA	2019
NR104826	CECT 4887	USA	2019
PQ732928	C5	China	2024
PQ732933	E2	China	2024
NR104901	P288	USA	2019
OR844313	seq G-3	China	2023

**Figure 3:**
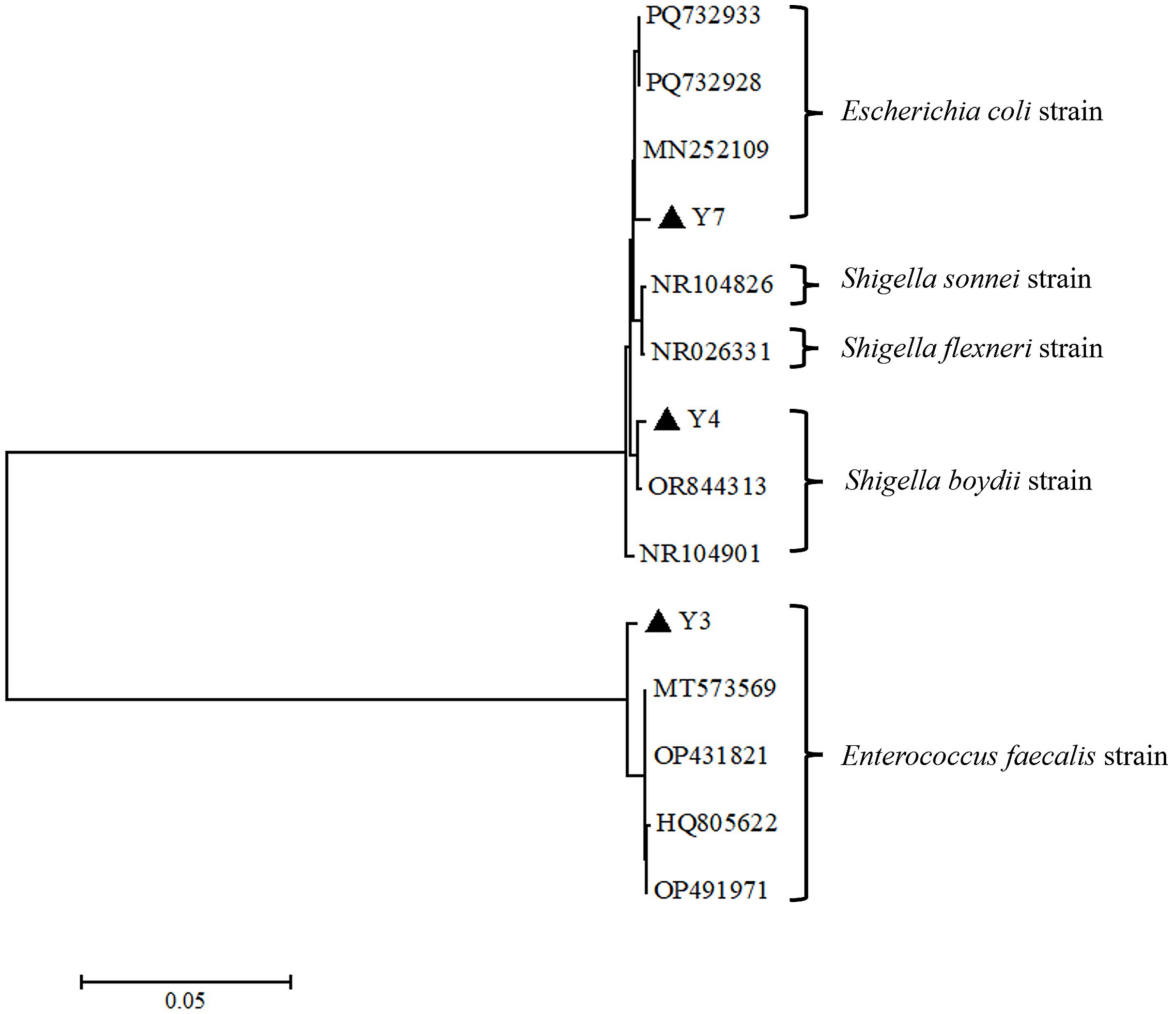
16S rRNA phylogenetic tree of isolated and reference bacteria (▲ indicates the target isolated strains).

The colonies on the plate were smooth—surfaced and off—white in color (Supplementary Figure 2). The outcomes of the agarose gel electrophoresis for the PCR products were depicted in Supplementary Figure 3. The size of the target band was estimated to be approximately 1500 bp. Subsequent to in—depth sequencing analysis, the isolated bacterial strains were conclusively identified as *Shigella boydii* (Y4), *Escherichia coli* (Y7), and *Enterococcus faecalis* (Y3). A 16S rRNA phylogenetic tree of the isolated strains was constructed (Figure 3). The resulting analysis demonstrated that the isolated strain Y3 was positioned within the same major clade as MT573569, OP431821, HQ805622, and OP491971, thus being taxonomically classified as *Enterococcus faecalis*. In addition, within this overall classification, strain Y3 also formed an independent minor clade and was determined to be of black—swan origin. Isolate Y7 was found to be part of the same major clade as MN252109, PQ732928, and PQ732933, and was accordingly assigned to the species *Escherichia coli*. Furthermore, isolate Y4 was located on the same clade as NR104901 and OR844313, and was classified as *Shigella boydii*. These bacteriological test results supported the pathological anatomical changes mentioned above, and further suggested the dead cause of the swan in the case.

## 5 Diagnosis

Based on the above clinical evidence, the cause of the black swan’s death in this case was determined as follows: The black swan was foraging in the water and ingested a long strip of cloth strip resembling a plant leaf. Due to the large size of the foreign object, it blocked the birds gizzard. As a result, the ingested sediment, food, and digestive juices secreted by the proventriculus of the bird could not enter the duodenum smoothly, and led to indigestion, nutritional deficiency, emaciation, and a decline in immunocompetence of the affected swan. Further it induced gastric perforation, which in turn gave rise to secondary bacterial infection (*Shigella boydii*, *Escherichia coli*, and *Enterococcus faecalis*), ultimately culminating in the death of the bird.

## 6 Discussion and conclusion

In the particularly clinical case, black swans kept in the campus lake with clean and pollution-free water, as well as good environment. All the 12 black swans, mainly inhabited in the central island of the lake, with no external interference around, and no other swans or poultry present (except for wild sparrows). The black swans were able to forage freely in the lake. Meanwhile, the keepers provided feed, which mainly consisted of corn bran and green vegetables. In this case, only one black swan died while the rest remained healthy. Before its death, the swan flock did not exhibit any symptoms of acute infectious diseases. Moreover, all the black swans had been vaccinated against gosling plague, paramyxovirus disease, and highly pathogenic influenza. The autopsy results showed that a cloth strip had blocked the digestive tract. At the same time, concurrence infection by pathogenic bacteria was also identified. Based on the above comprehensive analysis, the possibilities of acute infectious diseases and food poisoning could be excluded. Subsequent follow-up also showed that the remaining swans were normal, further supporting the clinical diagnosis. Before the autopsy, the author once suspected that it might be caused by a parasitic infection (manifested as emaciation, a protruding breastbone, and insufficient pectoral muscles) (6, 23). However, after the autopsy, it was discovered that the proventriculus of the dead black swan had perforated, and there was a large amount of sediment containing sands and a sizeable piece of foreign cloth in its gizzard, as well as no parasites were found in the intestines. Thus, the possibility of a parasitic infection was ruled out. The bacteriological results are consistent with the hypothesis of secondary infection, though sampling was limited to the liver. Therefore, the final diagnosis was made that the foreign cloth had blocked the proventriculus, causing a mixture of sediment and digestive juices to accumulate in gizzard. This brought about the perforation of the gastric wall, which in turn triggered a bacterial infection, ultimately resulting in the black swan’s death (3). This case is not only a special case in which a swan died of eating human waste cloth by mistake, but also a typical veterinary clinical teaching and practice, which enriches the clinical practice of clinical teachers and students.

In the case, the black swan that died inhabited either the lake or an artificial island. Prior to this, the “July 20 Extreme Rainstorm Incident” occurred in Zhengzhou, Henan Province.^1^ Due to these similar force majeure factors, human—generated waste, such as plastic products and non-degradable fabrics, may have entered into the lake. Additionally, strong winds also had inadvertently blown plastic bags into the lake. This type of undecomposed waste, such as submerged plant leaves, can be easily mistaken for food by birds in the lake, significantly impacting their health. The clinical case highlights that the waste we unintentionally discard in our daily lives can significantly affect the health of wildlife in the surrounding environment. For instance, on campus, staffs and students have occasionally seen a black swan picking up and nibbling on plastic bags (Supplementary Figure 4). This highlights the ongoing need for efforts in promoting environmental protection and wildlife conservation.

In addition, it is essential to improve the management of black swans in the campus lake, and increase the sense of responsibility among the caretakers. During daily inspections of black swans, staff should be responsible for observing the swans’ health conditions carefully so that the bird with abnormal actions would be found in a timely manner, it may avoid similar occurrence of black swan deaths. Furthermore, it is worth noting that the bird in this instance was frozen prior to autopsy and clinical examination, which might have affected the clinical observations to some extent.

## Data Availability

The datasets presented in this study can be found in online repositories. The names of the repository/repositories and accession number(s) can be found at: https://www.ncbi.nlm.nih.gov/genbank/, 2941539; https://www.ncbi.nlm.nih.gov/genbank/, 2941535; https://www.ncbi.nlm.nih.gov/genbank/, 2941509.

## Footnotes

^1^ https://www.mem.gov.cn/gk/sgcc/tbzdsgdcbg/202201/P020220121639049697767.pdf
