# Supplementary figures and images for: A case report of black swan (Cygnus atratus) died from gastric perforation and secondary infection resulting from ingestion of cloth—like foreign material
Source: Front Vet Sci. 2025 Sep 2;12:1608317. doi: 10.3389/fvets.2025.1608317 (PMC12441207; doi:10.3389/fvets.2025.1608317)

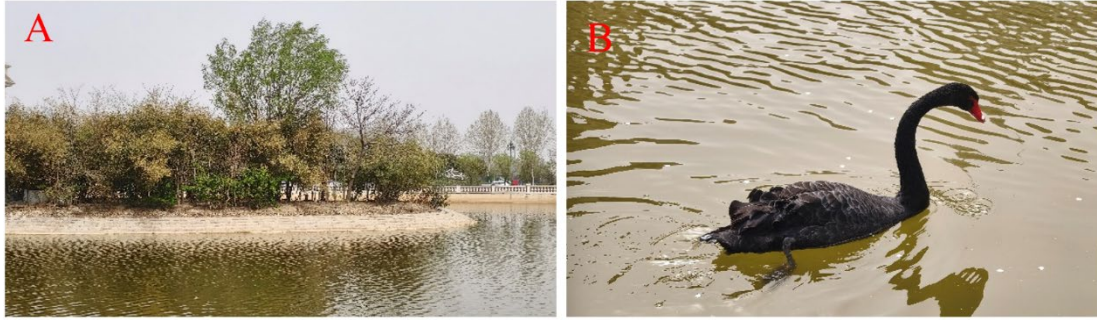

Supplement figure 1. Black swans and their habitats.A: Habitat of Black Swans; B: Normal Black Swans.

Supplement: Supplementary file 1 [file Image_1.pdf]

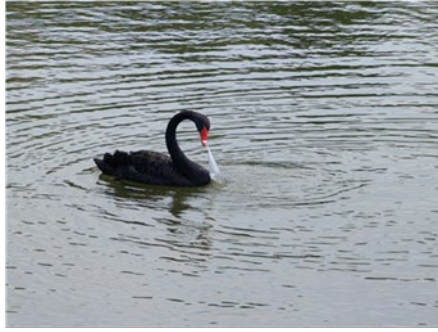

Supplement figure 4. A black swan was accidentally ingesting a plastic bag.

Supplement: Supplementary file 4 [file Image_4.pdf]
