# Supplementary material for: A case report of black swan (Cygnus atratus) died from gastric perforation and secondary infection resulting from ingestion of cloth—like foreign material
Source: Front Vet Sci. 2025 Sep 2;12:1608317. doi: 10.3389/fvets.2025.1608317 (PMC12441207; doi:10.3389/fvets.2025.1608317)
Supplement: Supplementary file 2 [file Image_2.pdf]

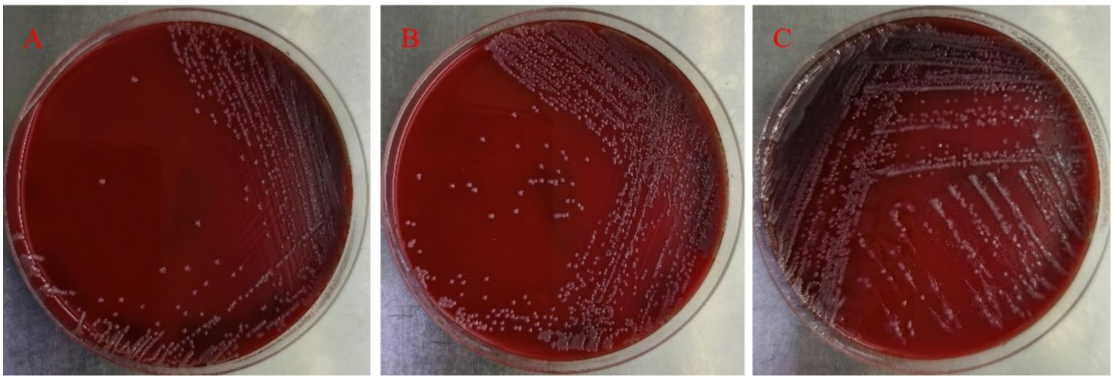

Supplement figure 2. Growth of single bacteria on the blood - agar plate. A: Isolated Strain Y4; B: Isolated Strain Y7; C: Isolated Strain Y3.
