# Supplementary material for: A case report of black swan (Cygnus atratus) died from gastric perforation and secondary infection resulting from ingestion of cloth—like foreign material
Source: Front Vet Sci. 2025 Sep 2;12:1608317. doi: 10.3389/fvets.2025.1608317 (PMC12441207; doi:10.3389/fvets.2025.1608317)
Supplement: Supplementary file 3 [file Image_3.pdf]

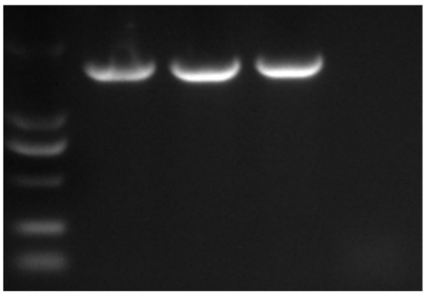

Supplement figure 3. Results of PCR Gel Electrophoresis. M: 2000 DNA Marker; 1: Isolated Strain Y7; 2: Isolated Strain Y3; 3: Isolated Strain Y4; 0: Negative Control.
